# Selection and Validation of Housekeeping Genes as Reference for Gene Expression Studies in Pigeonpea (*Cajanus cajan*) under Heat and Salt Stress Conditions

**DOI:** 10.3389/fpls.2015.01071

**Published:** 2015-12-21

**Authors:** Pallavi Sinha, Rachit K. Saxena, Vikas K. Singh, L. Krishnamurthy, Rajeev K. Varshney

**Affiliations:** ^1^Applied Genomics, Centre of Excellence in Genomics, International Crops Research Institute for the Semi-Arid TropicsHyderabad, India; ^2^School of Plant Biology and Institute of Agriculture, The University of Western Australia, PerthWA, Australia

**Keywords:** heat stress, salt stress, quantitative real-time PCR, housekeeping genes

## Abstract

To identify stable housekeeping genes as a reference for expression analysis under heat and salt stress conditions in pigeonpea, the relative expression variation for 10 commonly used housekeeping genes (*EF1α, UBQ10, GAPDH, 18Sr RNA, 25Sr RNA, TUB6, ACT1, IF4α, UBC, and HSP90*) was studied in root, stem, and leaves tissues of Asha (ICPL 87119), a leading pigeonpea variety. Three statistical algorithms geNorm, NormFinder, and BestKeeper were used to define the stability of candidate genes. Under heat stress, *UBC*, *HSP90*, and *GAPDH* were found to be the most stable reference genes. In the case of salinity stress, *GAPDH* followed by *UBC* and *HSP90* were identified to be the most stable reference genes. Subsequently, the above identified genes were validated using qRT-PCR based gene expression analysis of two universal stress-resposive genes namely *uspA* and *uspB*. The relative quantification of these two genes varied according to the internal controls (most stable, least stable, and combination of most stable and least stable housekeeping genes) and thus confirmed the choice as well as validation of internal controls in such experiments. The identified and validated housekeeping genes will facilitate gene expression studies under heat and salt stress conditions in pigeonpea.

## Introduction

Pigeonpea, one of the major food legume of tropic and sub-tropic, encounters various abiotic stresses during its life cycle (Varshney et al., 2012). As a rain-fed growing crop, among different abiotic stresses, moisture stress is more prevalent during various stages of the life cycle in pigeonpea (Choudhary et al., 2011). For instance, in the north-western part of India, extreme high temperature (heat stress) during reproductive stage had hampered the crop, leading to severe yield loss (Choudhary et al., 2011). Similarly, accumulation of the excess amount of salt in the soil surface is very harmful and could result in the damage in plant growth by interfering with the mineral nutrient uptake (Chikelu et al., 2007). It has also been shown that higher salt concentration reduced important agronomic traits like, plant height, leaf area, crop growth rate, total dry matter, net assimilation rate, and seed yield, etc. (Joshi and Nimbalkar, 1983).

In view of above, there is a demand of development of high yielding and multiple stresses resistant varieties in pigoenpea (Pazhamala et al., 2015). Availability of draft genome sequence has opened an unprecedented opportunity to investigate the genetic basis of abiotic stress resistance in pigeonpea (Varshney et al., 2012). For instance, it has become possible now to identify candidate genes either by mining directly from the pigeonpea genome or identifying the homo-/ortho-logous genes for the candidate genes identified in other crop species. qRT-PCR is one of the most robust and reliable techniques of gene expression studues. For an accurate measurement and reproducible expression profiling of target genes in qRT-PCR analysis, use of stable housekeeping genes, also called as ‘internal control’ is essential to normalize the expression level. Housekeeping genes work for the basic cellular and metabolic functions and maintains the stable and constitutive expression throughout, irrespective of any external physiological conditions (Yang et al., 2014). However, several reports available across species stated that the expressions of housekeeping genes may vary depending on different external factors (Greer et al., 2010; Liu et al., 2012; Duhoux and Délye, 2013). The selection of a suitable housekeeping genes to normalize the expression level is a challenging task and requires extensive study to get an accurate result (Wang et al., 2015). For instance, expression of commonly used reference genes, i.e., *ACT1* and *GAPDH* has been found varying across different tissues, developmental stages, and different experimental conditions (Fischer et al., 2005; Goossens et al., 2005; Brinkhof et al., 2006; Sinha et al., 2015). Therefore, for consistent and reliable results, housekeeping genes should be chosen and validated prudently (Wang et al., 2015). Additionally, a combination of multiple numbers of reference genes will give more precision, where the geometrical mean of multiple internal controls will minimize the expressional variation (Vandesompele et al., 2002). In the case of pigeonpea, *IF4α* and *TUB6* genes had recently been identified as stable housekeeping genes for undertaking gene expression studies under drought stress conditions in pigeonpea (Sinha et al., 2015).

Keeping in view of above, the present study reports identification of the most stable gene(s) for gene expression studies under heat and salt stress conditions. These genes are expected to accelerate gene expression studies especially for heat and salt stresses in pigeonpea.

## Materials and Methods

### Plant Material and Growth Conditions

For the gene expression analysis, ICPL 87119 (Asha), a medium duration, high yielding variety was selected. Genetically pure seeds, developed by crossing C11 × ICP1-6-W3/W, were collected from Pigeonpea Breeding Division, ICRISAT, Patancheru. Seeds were surface sterilized with sodium hypochlorite, thoroughly washed with DEPC treated water and pre-soaked overnight. Germinated seedlings were sown in a three inch plastic pots (one per pot) filled with autoclaved black soil, sand, and vermicompost (10:10:1 v/v) mixture. Fresh root, shoot and leaf tissues were harvested from all the pots, immediately frozen in liquid nitrogen and stored in -80 deep freezer till RNA isolation.

### Heat and Salt Stress Treatments

For heat stress, 45-days-old (vegetative stage) and 75-days-old-plants (reproductive stage) were transferred from glass-house to growth chamber (12 h/12 h light/dark), 32°C/20°C day/night and 50% relative humidity (RH) whereas control plants were maintained at normal glass-house conditions. The saline solution was added on 7-days-old seedlings (vegetative stage) and 75-days-old-plants (reproductive stage) for salt stress. Total of 120 mM NaCl solution was added to stress plants and tissues were harvested after 5 days of stress treatment.

### RNA Isolation

Total RNA was isolated using TRIzol reagent (Invitrogen, USA) and purified using DNase (Qiagen, GmbH, Germany) through an RNeasy Plant Mini kit according to the manufacturer’s instruction. The integrity of isolated RNA was checked on 0.8% agarose/formaldehyde (FA) gel electrophoresis. The concentration of each sample was checked on the Qubit fluorometer (Invitrogen) and three micrograms of RNA was used for first-strand cDNA synthesis using the SuperScript^®^III RT enzyme (Invitrogen, USA) following the manufacturer’s guidelines.

### Selection of Housekeeping Genes

Based on various gene expression studies in different crops, a set of 10 genes namely *EF1α, UBQ10, GAPDH, 18Sr RNA, 25Sr RNA, TUB6, ACT1, IF4α, UBC*, and *HSP90* were selected. Details of these genes have been provided in **Table 1**. These genes were subjected to homology search in pigeonpea genome, and their homologs were used for primer designing. The amplicon size ranged from 95 bp for *GAPDH* and *IF4α* genes to 107 bp for *25Sr RNA*.

**Table 1:** Details on primers used for qRT-PCR analysis.

Gene name	Gene ID	Primer sequence (5′–3′)	Amplicon size (bp)	qRT- PCR efficiency (%)	Correlation coefficient (*R*^2^)
*EF1α*	B9SPV9	F-GAGAGGTCCACCAACCTTGA	103	103.09	0.9995
		R-TTGTAGACGTCCTGCAATGG			
*UBQ10*	Q8H159	F-CCAGACCAGCAGAGGTTGAT	102	104.43	0.9991
		R-GATCTGCATACCTCCCCTCA			
*GAPDH*	Q2I0H4	F-ATGGCATTCCGTGTTCCTAC	95	99.66	0.9996
		R-CCTTCAACTTGCCCTCTGAC			
*18Sr RNA*	A5COJ4	F-CCACTTATCCTACACCTCTC	102	104.43	0.9951
		R-ACTGTCCCTGTCTACTATCC			
*25Sr RNA*	B7FKH8	F-ACCCTTTTGTTCCACACGAG	107	97.23	0.9965
		R-GACATTGTCAGGTGGGGAGT			
*TUB6*	B9R897	F-GCCCTGACAACTTCGTCTTC	100	103.98	0.9878
		R-GCAGTTTTCAGCCTCTTTGC			
*ACT1*	C6TJ78	F-GGCATACATTGCCCTTGACT	97	100.92	0.9984
		R-GAACCTCGGGACATCTGAAA			
*IF4α*	C6T8X3	F-GCCGAGATCACACAGTCTCA	95	90.94	0.9985
		R-ACCACGAGCCAAAAGATCAG			
*UBC*	Q2V732	F-CGAGAAAAGGCAGTTGATCC	105	104.43	0.9988
		R-CAGAAAAGGCAAGCTGGAAC			
*HSP90*	A5AHA8	F-TGTCGAGCAAGAAGACGATG	103	100.50	0.9975
		R-GGGCAGTTTCAAAGAGCAAG			


### Primer Designing and Quantitative Real-time PCR

Ten commonly known housekeeping genes, listed in **Table 1** were subjected to get pigeonpea orthologous sequences and used for primer designing. The functional integrity of the obtained sequences were checked using BLASTN search against GenBank EST database^1^ (IIPG). Primer pairs were designed from exonic regions using Primer3Plus software^2^.

The qRT-PCR was carried out using ABI SYBR^®^ GREEN PCR reaction on an ABI Fast7500 System [Applied Biosystems (ABI), Foster City, CA, USA] according to the manufacturer’s instructions. The amplification efficiency of primers was estimated by SYBR Green chemistry RT-qPCR (Sinha et al., 2015). PCR conditions for all the qRT-PCR reactions were used as followings: 2 min at 50°C, 10 min at 95°C, and 40 cycles of 15 s at 95°C and 1 min at 60°C. Each reaction was performed in three biological and two technical replicates along with no template control. Melting curve analysis and agarose gel electrophoresis were carried out to check the amplicon specificity.

### Gene Expression Analysis

Gene expression stability of 10 selected housekeeping genes in the root, shoot, and leaf tissues under heat and salt stress conditions was determined by BestKeeper descriptive statistical tool (Pfaﬄ et al., 2004). The tool is a Microsoft Excel based, freely downloadable software^3^ that identifies the most suitable reference gene by repeated pairwise correlation and regression analysis of each gene with the other remaining candidate reference genes.

For ranking and identification of the most stable housekeeping genes for given conditions, statistical algorithms geNorm and NormFinder were used. The geNorm^4^ algorithm measures the average expression stability value (*M-*value) and identifies two most stable genes from the analysis (Vandesompele et al., 2002). NormFinder is a Microsoft Excel based program^5^ that works on the linear mixed-effects modeling to calculate stability values. The programe identifies the optimum number of housekeeping genes to be used in normalization studies for qRT-PCR analysis (Andersen et al., 2004).

### Validation of Identified Reference Genes

The most stable housekeeping genes, identified during the present study were validated in the root, shoot and leaves tissues from heat and salt-stressed conditions. Two previously identified drought responsive universal stress protein coding genes, *uspA* and *uspB* (data unpublished) were used to validate the most stable, combination of most stable, least stable, and commonly used housekeeping genes. The differential gene expression of heat and salt stressed samples were compared to their respective unstressed controls with respect to different reference genes using a Relative Expression Software Tool (REST©) (Pfaﬄ et al., 2002).

## Results

### Expression Profiling of Housekeeping Genes

**FIGURE 1:**
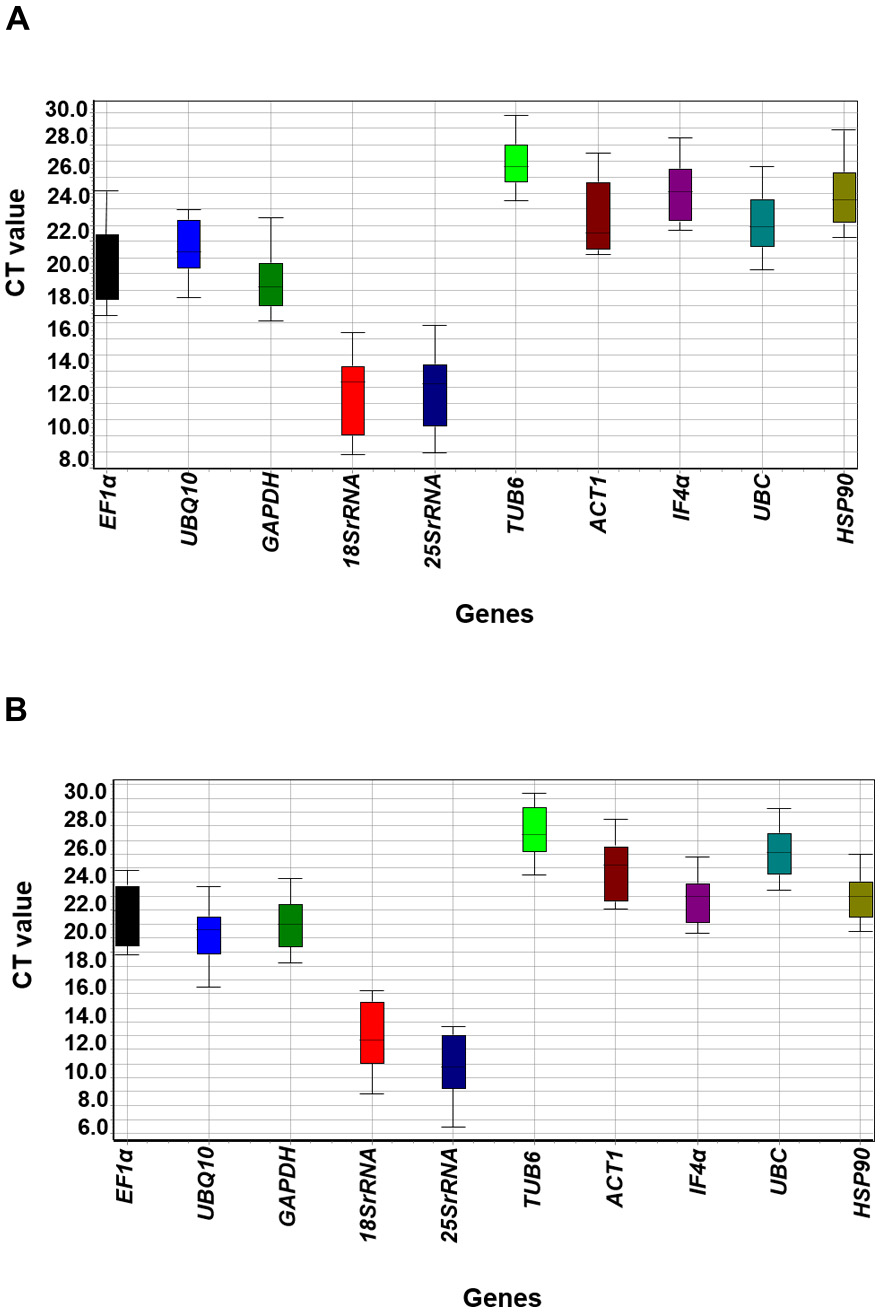
**Ct variation of tested housekeeping genes among different tissue samples under heat and salt stresses.** **(A)** Boxplot depicting absolute Ct values of heat imposed/control samples and **(B)** Boxplot depicting absolute Ct values of salt imposed/control samples. Lower and upper boxes indicate the 25th and 75th percentile, respectively. The median is depicted by the horizontal line in the box.

To identify the most stable housekeeping genes, mRNA levels in all 24 tissues (stress imposed and control) were quantified based on their cDNA concentration. Detailed information on these 24 tissue samples has been given in **Supplementary Table S1**. The PCR efficiencies of each of the primers used in the present study were calculated based on 10-fold serial dilutions of pooled cDNA as reported previously (Sinha et al., 2015). The qRT-PCR efficiency (%) ranged from 90.94 (I*F4*α) to 104.43 (*UBQ10, 18Sr RNA*, and *UBC*) (**Table 1**). The obtained results were in accordance to the Minimum Information for Publication of Quantitative Real-Time PCR Experiments (MIQE) guidelines, the ideal PCR efficiency is 100%, while the acceptable range is from 80 to 120% (Bustin et al., 2009). The mean cycle threshold (Ct) values of all the 10 candidate genes for 12 different samples of heat ranged from *7.8 (18Sr RNA* in LHRS*)* to *28.8 (TUB6* in EHSC*)*. Similarly for salt stress conditions, the mean Ct values ranged from 5.4 (*25Sr RNA* in LHRC) to 29.3 (*TUB6* in ESRC) (**Figure 1** and **Supplementary Figure S1**). Further, to define the ranking of targeted housekeeping genes for heat as well as salt stress conditions, three different algorithms namely BestKeeper, geNorm, and NormFinder were used as given in section below.

### Identification of Suitable Reference Genes for Heat Stress Conditions

Descriptive statistics for each gene across tissues was estimated using BestKeeper algorithm. The analysis determined standard deviation (SD) value with ≤1 for all targeted housekeeping genes, indicating their consistent and stable performance (**Supplementary Table S2**). This analysis revealed that *TUB6* (SD, 1.13) showed the least SD followed by *UBQ10* (SD, 1.45) and *GAPDH* (SD, 1.49). Two genes namely *25Sr RNA* (SD, 2.08) and *18Sr RNA* (SD, 2.08), followed by *EF1α* (SD, 2.04) showed higher SD during the analysis reflecting their unstable nature under heat stress conditions. The coefficient of variations (CVs) of all the tested housekeeping genes ranged from 4.39 for *TUB6* to 17.83 for *18Sr RNA*.

**FIGURE 2:**
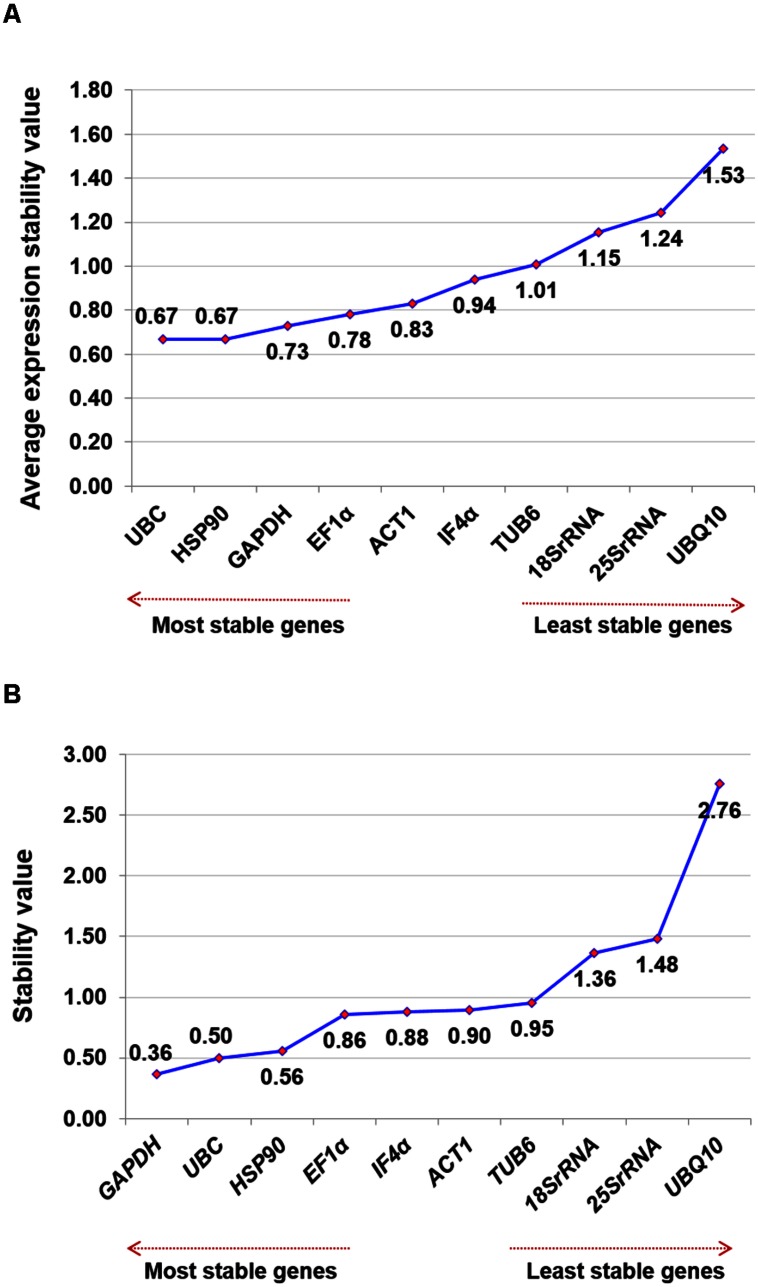
**Ranking of housekeeping genes for heat stress conditions.** Gene expression studies for identification of most stable housekeeping genes under heat stress condition using two software programs. The direction of arrow indicates the most and least stable housekeeping genes in graphs **(A)** Gene expression stability graph of housekeeping gene using geNorm program based on average expression stability value (*M*), which is based on stepwise exclusion process. *M*-value is inversely related to gene stability **(B)** Gene expression stability graph using NormFinder program based on stability value and lower the stability value indicates higher stability of the housekeeping genes.

To determine the ranking of selected housekeeping genes based on average expression stability value (*M-*value), geNorm algorithm was used. geNorm analysis of datasets revealed *UBC* and *HSP90* (*M*-value of 0.666) were the best pair of stable genes on the basis of their average expression stability value followed by *GAPDH* (*M*-value of 0.728) and *EF1α* (*M*-value of 0.780) (**Table 2**). Based on *M*-value, *18Sr RNA* (*M*-value of 1.154), *25Sr RNA* (*M*-value of 1.244) and *UBQ10* (*M*-value of 1.532) were found to be the least stable genes for expression studies. All 10 tested genes showed relatively high stability with *M*-value of less than 1.5 except *UBQ10*, indicating that genes used in the present study performed stable under heat stress conditions. Graphical representation of all the selected housekeeping genes are illustrated in **Figure 2A**.

**Table 2:** Ranking of tested housekeeping genes for heat stress conditions using geNorm and NormFinder algorithms.

Factor	geNorm		NormFinder	
		
	*M-*value	Ranking	Stability value	Ranking
*EF1α*	0.780	3	0.858	4
*UBQ10*	1.532	9	2.755	10
*GAPDH*	0.728	2	0.362	1
*18Sr RNA*	1.154	7	1.364	8
*25Sr RNA*	1.244	8	1.481	9
*TUB6*	1.007	6	0.951	7
*ACT1*	0.831	4	0.896	6
*IF4*α	0.938	5	0.879	5
*UBC*	0.666	1	0.496	2
*HSP90*	0.666	1	0.558	3


In addition to above mentioned algorithms, NormFinder analysis was also used to identify the most stable genes, on the basis of stability value. Based on this analysis, *GAPDH* (stability value, 0.362), *UBC* (stability value, 0.496) and *HSP90* (stability value, 0.558) were identified as the most stable reference gene. Similar to the results obtained from geNorm analysis, *18Sr RNA* (stability value, 1.364), *25Sr RNA* (stability value, 1.481), and *UBQ10* (stability value, 2.755) were found as the least stable genes for heat stress conditions (**Figure 2B**) in the NormFinder analysis.

Although marginal differences were observed in the overall ranking of all the candidate genes tested in the present study, comparative analysis based on the geNorm and NormFinder output results showed that *UBC*, *HSP90* followed by *GAPDH* are the three most stable genes (**Table 2** and **Figures 2A,B**). The present findings were further supported by the heat map of the individual genes based on the Ct values, which correlates the stability ranking of the identified genes (**Supplementary Figure S2**).

### Identification of Suitable Reference Genes for Salt Stress Conditions

For identification of the most appropriate reference gene under salinity stress, 10 housekeeping genes were analyzed in 12 different tissues (salinity imposed and control tissues). The descriptive analysis of datasets of all tested housekeeping genes under salinity stress conditions were determined by BestKeeper (**Supplementary Table S2**). Based on the SD value, *IF4α* (SD, 1.44) was identified as the most stable gene followed by *HSP90* (SD, 1.46) and *TUB6* (SD, 1.47). However, *EF1*α (SD, 1.85), *25Sr RNA* (SD, 1.86), and *18Sr RNA* (SD, 2.06) were found as the least stable genes under salt stress condition. The CV of all the tested genes were found higher and ranged from 5.56 for *TUB6* and 19.27 for *25Sr RNA*.

**FIGURE 3:**
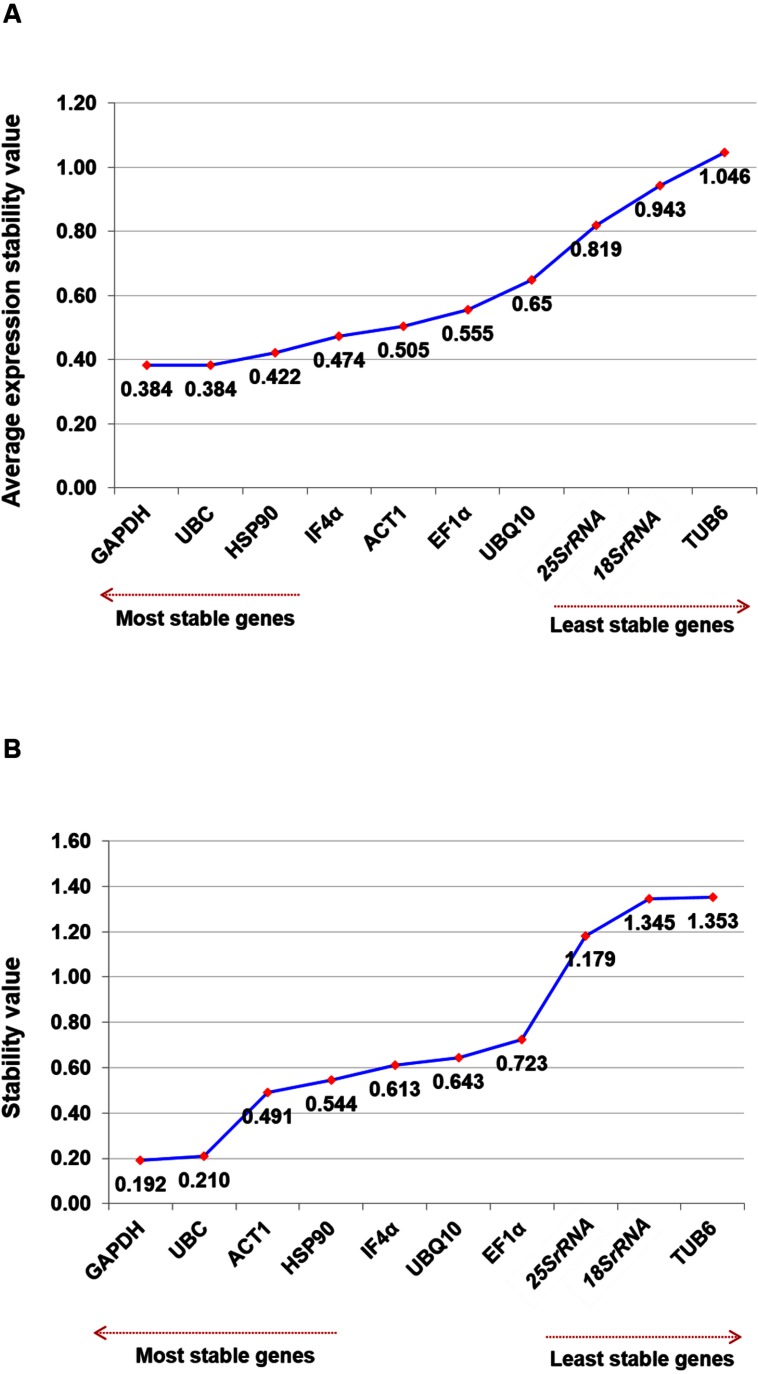
**Ranking of housekeeping genes for salinity stress conditions.** Gene expression studies for identification of most stable housekeeping genes under salt stress condition using two software programs. The direction of arrow indicates the most and least stable housekeeping genes in graphs **(A)** Gene expression stability graph of housekeeping gene using geNorm program based on an average expression stability value (*M*), which is based on stepwise exclusion process. *M*-value is inversely related to gene stability **(B)** Gene expression stability graph using NormFinder program based on stability value and lower the stability value indicates higher stability of the housekeeping genes.

The two most stable housekeeping genes, namely *GAPDH* and *UBC* (*M*-value, 0.384) were identified using geNorm analysis for salinity stress conditions followed by *HSP90* (*M*-value, 0.422) and *IF4α* (*M*-value, 0.474). However, *TUB6* (*M*-value, 1.046) was found as the least stable gene compared to all other tested genes (**Figure 3A**). Even though on the basis of *M-*value, all of the tested genes showed relatively high stability with *M*-value of less than 1.5. Overall ranking of all tested genes using both the software for the salt stress condition is presented in **Table 3**.

**Table 3:** Ranking of tested housekeeping genes for salt stress conditions using geNorm and NormFinder algorithms.

Factor	geNorm		NormFinder	
		
	*M-value*	Ranking	Stability value	Ranking
*EF1α*	0.555	5	0.723	7
*UBQ10*	0.650	6	0.643	6
*GAPDH*	0.384	1	0.192	1
*18Sr RNA*	0.943	8	1.345	9
*25Sr RNA*	0.819	7	1.179	8
*TUB6*	1.046	9	1.353	10
*ACT1*	0.505	4	0.491	3
*IF4α*	0.474	3	0.613	5
*UBC*	0.384	1	0.210	2
*HSP90*	0.422	2	0.544	4


NormFinder analysis of the datasets identified *GAPDH* (stability value, 0.192) as the most stable gene followed by *UBC* (stability value, 0.210) and *ACT1* (stability value, 0.491) (**Figure 3B**). Based on the geNorm and NormFinder analysis, *GAPDH* was ranked as the most stable gene followed by *UBC* and *HSP90*. Similar to other stress conditions, *25Sr RNA* (stability value, 1.179) and *18Sr RNA* (stability value, 1.345) genes were found as the two least stable genes (**Table 3**). Additionally, using the Ct values, heat map was generated for all the candidate genes tested across the tissues. The heat map analysis revealed the stable level of expression of *GAPDH*, across the tissues and stages (**Supplementary Figure S2**).

### Validation of Identified Stable Reference Genes for Heat Stress Conditions

**FIGURE 4:**
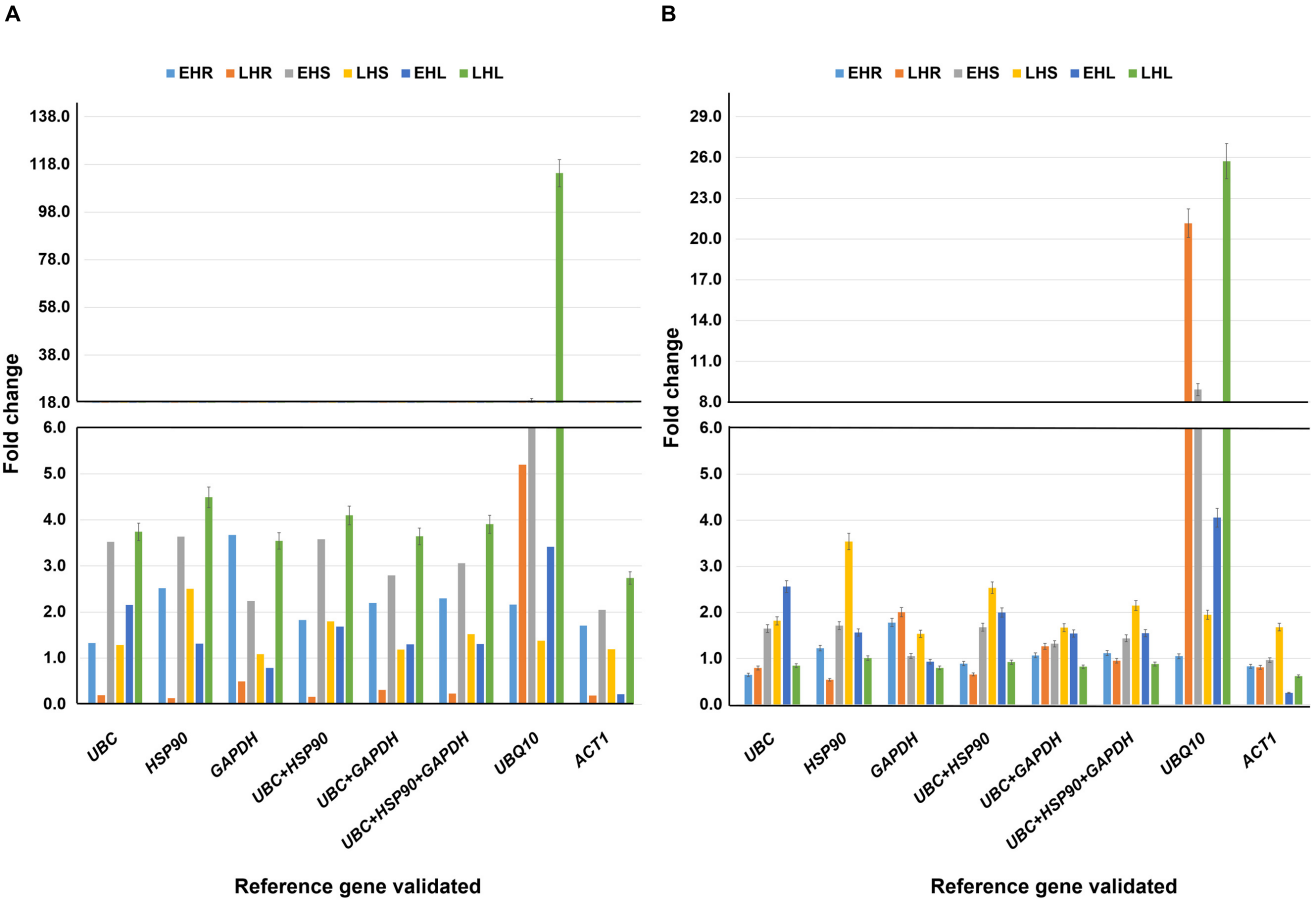
**Validation of reference genes under heat stress conditions.** Expression profiling of candidate gene **(A)** *uspA* and **(B)** *uspB* in heat imposed tissues (root, stem, and leaves) and normalized with (i) *UBC* (ii) *HSP90* (iii) *GAPDH* (iv) *UBC* + *HSP90* (v) *UBC* + *GAPDH* (vi) *UBC* + *HSP90* + *GAPDH* (vii) *UBQ10* and (viii) *ACT1*. The analysis was completed in two different stages with six different tissues. The relative quantification values of selected drought responsive candidate gene were obtained after scaling to control samples. EHR, vegetative root stressed; LHR, reproductive root stressed; EHS, vegetative stem stressed; LHS, reproductive stem stressed; EHL, vegetative leaves stressed; LHL, reproductive leaves stressed.

To test the performance of identified most stable housekeeping genes, two earlier identified universal stress protein genes namely, *uspA* and *uspB* were used as target genes. Three most stable housekeeping genes identified in the present study (*UBC, HSP90*, and *GAPDH*), their combinations (*UBC* + *HSP90*, *UBC* + *GAPDH*, and *UBC* + *HSP90* + *GAPDH*), most commonly used housekeeping gene (*ACT1*) and least stable (*UBQ10*) genes were used as internal controls. The expression analysis was performed in three different tissues (root, stem, and leaf) at early and late heat stress conditions. As a result, varied level of expression differences was observed for both the target genes while normalized with different internal controls (**Figures 4A,B**).

For *uspA* gene, late heat root (LHR) tissues showed higher level of expression with *UBQ10* (5.20 fold) as compared to the stable *UBC* (0.20 fold), *HSP90* (0.13 fold), *GAPDH* (0.49 fold), and combination of stable genes, *UBC* + *HSP90* (0.16 fold), *UBC* + *GAPDH* (0.31 fold) and *UBC* + *HSP90* + *GAPDH* (0.23 fold). Simillary, *uspA* gene in early heat stem (EHS) and late heat leaf (LHL) tissues showed very high level of gene expression with *UBQ10* as reference gene (18.94 fold for EHS and 114.30 fold for LHL). The expression *of uspA* in EHS and LHL varied when we used different stable reference genes, e.g., *UBC* (3.52 fold for EHS and 3.74 fold for LHL), *HSP90* (3.64 fold for EHS and 4.49 fold for LHL), *GAPDH* (2.24 fold for EHS and 3.55 fold for LHL) and combinations of different stable reference genes such as *UBC* + *HSP90* (3.58 fold for EHS and 4.10 fold for LHL), *UBC* + *GAPDH* (2.80 fold for EHS and 3.64 fold for LHL) and *UBC* + *HSP90* + *GAPDH* (3.06 fold for EHS and 3.91 fold for LHL) (**Figure 4A**).

In the case of *uspB* gene, *UBQ10* in comparison to stable and combination of stable genes showed higher gene expression in LHR and LHL tissues. The gene expression of *UBQ10* was 21.16 fold for LHR and 25.73 fold for LHL. For stable genes, *UBC* showed 0.80 fold in LHR and 0.84 fold gene expression in LHL tissues, *HSP90* showed 0.54 fold in LHR and 1.01 fold in LHL, *GAPDH* showed 2.01 fold in LHL and 0.80 fold in LHL tissues. Similarly, the combinations of stable genes, *UBC* + *HSP90* (0.65 fold in LHR and 0.92 fold in LHL tissues), *UBC* + *GAPDH* (1.26 fold in LHR and 0.82 fold in LHL tissues) and *UBC* + *HSP90* + *GAPDH* (0.95 fold in LHR and 0.88 fold in LHL tissues) showed similar level of expression as of stable genes (**Figure 4B**).

### Validation of Identified Stable Reference Genes for Salt Stress Conditions

**FIGURE 5:**
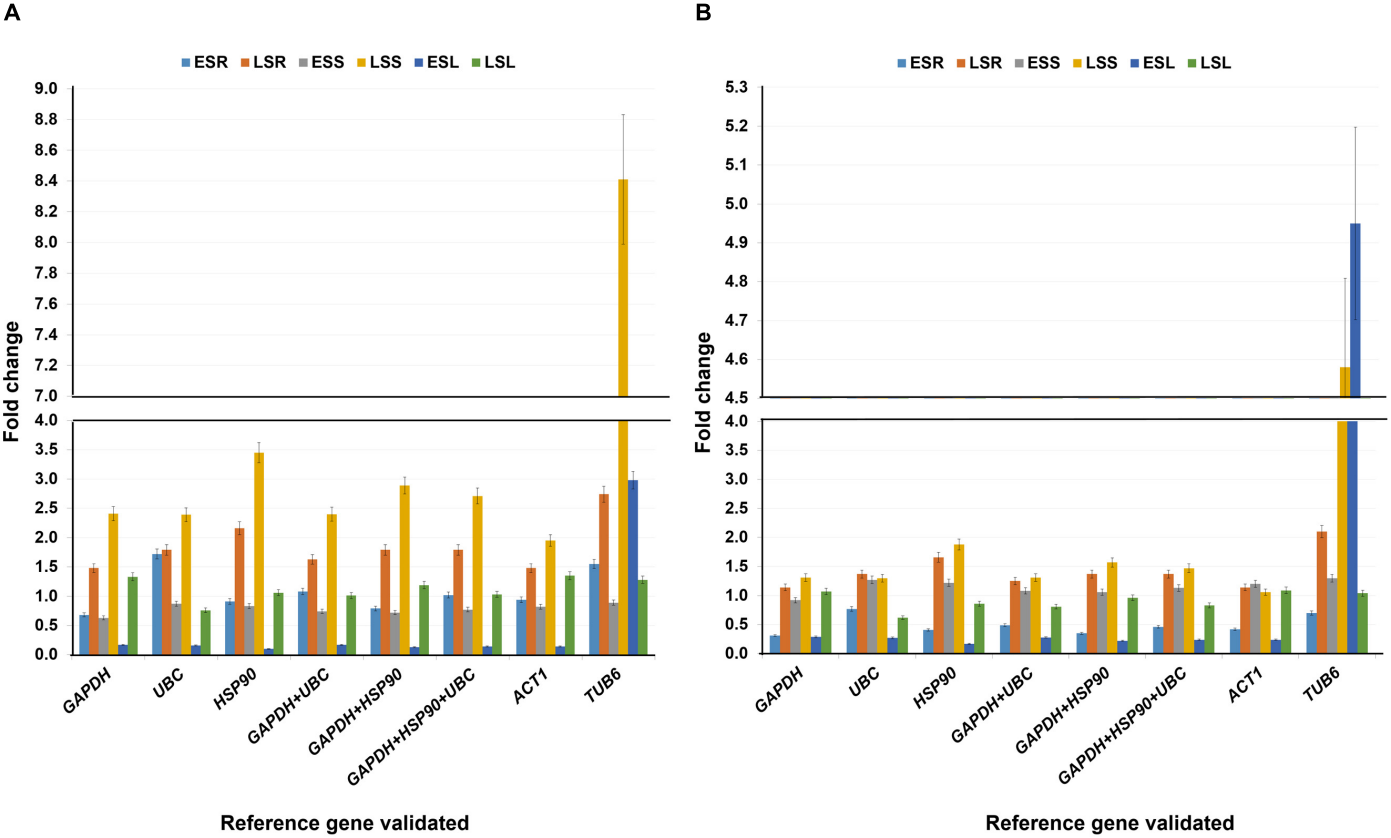
**Validation of reference genes under salt stress conditions.** Expression profiling of candidate gene **(A)** *uspA* and **(B)** *uspB* in salt imposed tissues (root, stem, and leaves) and normalized with (1) *GAPDH* (ii) *UBC* (iii) *HSP90* (iv) *GAPDH* + *UBC* (v) *GAPDH* + *HSP90* (vi) *GAPDH* + *HSP90* + *UBC* (vii) *ACT1* and (viii) *TUB6*. The analysis was completed in two different stages with six different tissues. The relative quantification values of selected drought responsive candidate gene were obtained after scaling to control samples. ESR, vegetative root stressed; LSR, reproductive root stressed; ESS, vegetative stem stressed; LSS, reproductive stem stressed; ESL, vegetative leaves stressed; LSL, reproductive leaves stressed.

The identified most stable housekeeping genes for salt stress conditions were also validated with previously identified two universal stress protein genes namely, *uspA* and *uspB.* Three most stable housekeeping genes (*GAPDH, UBC*, and *HSP90*), combination of stable genes (*GAPDH* + *UBC*, *GAPDH* + *HSP90* and *GAPDH* + *UBC* + *HSP90*) along with the most commonly used (*ACT1*) and the least stable housekeeping gene (*TUB6*) identified during the present study were used as internal control (**Figures 5A,B**).

The relative expression of the target gene *uspA* under salt stress conditions were almost similar with all the tested reference genes or combinations. However, the least stable housekeeping gene, *TUB6* showed a different expression pattern with a very high gene expression value. Briefly, *TUB6* gene showed showed 8.41 fold gene expression in late salt shoot (LSS) tissues. However, with stable genes the expression was 2.41 fold (*GAPDH*), 2.39 fold (*UBC*), 3.45 fold (*HSP90*), 2.40 fold (*GAPDH* + *UBC*), 2.89 fold (*GAPDH* + *HSP90*), and 2.71 fold (*GAPDH* + *UBC* + *HSP90*) in the same tissue. Similarly, in the early salt leaf (ESL) tissues, *GAPDH* showed 0.17 fold, UBC 0.16 fold, HSP90 0.10 fold, *GAPDH* + *UBC 0.17* fold, *GAPDH* + *HSP90 0.13* fold, and *GAPDH* + *UBC* + *HSP90* 0.14 fold gene expression, which was very low as compared to *TUB6* with 2.98 fold expression (**Figure 5A**).

The *uspB* gene expression in the LSS and ESL also showed a similar pattern found with the *uspA* gene, during the validation studies. The expression level of *uspB* gene in LSS tissue was checked in presence of reference genes, e.g., *GAPDH* (1.31 fold), *UBC* (1.30 fold), *HSP90* (1.88 fold), and *TUB6* (4.58 folds) as well as combinations of different reference genes, such as *GAPDH* + *UBC* (1.31 fold), *GAPDH* + *HSP90* (1.57 fold)*, GAPDH* + *UBC* + *HSP90* (1.47 fold). Following the same pattern, ESL tissue showed expression of 0.29 fold (*GAPDH*), 0.27 fold (*UBC*), 0.17 fold (*HSP90*), 0.28 fold (*GAPDH* + *UBC*), 0.22 fold (*GAPDH* + *HSP90*), and 0.24 fold (*GAPDH* + *UBC* + *HSP90*) in comparison to *TUB6* with a higher gene expression (4.95 fold) (**Figure 5B**).

## Discussion

For better understanding of regulation and function of genes involved in different stresses, it is pre-requisite to perform quantitative measurements and determine gene regulation patterns between samples (Van Hiel et al., 2009). To determine accurate measurement of target candidate gene(s), selection of a suitable reference gene, is pre-requisite during expression studies. An inappropriate reference gene can entirely change the base reference leading to an incorrect result interpretation (Dheda et al., 2005). Despite the fact that housekeeping genes exhibits no or minimum expression variations, many studies have proven the fact that such a perfect housekeeping gene has not yet reported which can be used as reference across different stress conditions (Zhu et al., 2013; Lopez-Pardo et al., 2013; Yang et al., 2014). Therefore, reference genes must be validated for each experimental condition in different species (Schmittgen and Zakrajsek, 2000).

To select appropriate reference genes for heat and salinity stress conditions, we have analyzed 10 commonly used housekeeping genes in a set of 24 diverse tissues (12 for each stress conditions) in pigeonpea. Three programs namely, BestKeeper, geNorm, and NormFinder were used to find out the stable housekeeping gene(s) in the given sample set and experimental design for different stress conditions. BestKeeper determines the optimal housekeeping gene employing the *pair-wise correlation analysis* of all pair of candidate genes (Pfaﬄ et al., 2004). Another program, geNorm works upon stepwise exclusion of the least stable genes, based on the average expression stability (*M*) value and which is indirectly proportional to stability of genes, i.e., lower the *M*-value higher the stability of genes (Vandesompele et al., 2002). The geNorm algorithm provides a pair of ideal housekeeping gene with identical expression ratios. NormFinder is an Excel based algorithm for identification of most stable gene based on the expression stability value (Andersen et al., 2004). As the three programs work on three different algorithms, they may provide different results (Mallona et al., 2010; Mafra et al., 2012; Zhu et al., 2013). Based on previous studies and algorithm they work upon, we have utilized BestKeeper for analyzing descriptive studies of different housekeeping genes and geNorm and NormFinder were used to determine the ranking of genes used in the present study.

The BestKeeper software provides two measures that can be used for assessing the stability of the reference genes. (i) raw SD of the Cq values and (ii) geometric mean of the reference genes and performs Pearson correlation of each of the reference genes to the BestKeeper Index. In the case of geNorm algorithms pairwise correlation known to be a strong algorithm for small sample sizes, but is biased toward selecting genes that are mutually correlated. Similarly, NormFinder has the strength that it can differentiate intragroup variation from intergroup variations. This software is useful for identifying candidate genes when different sample groups are to be compared. Therefore, differences among the underlying algorithms of three software packages are difficult for direct comparison among them. Recently, De Spiegelaere et al. (2015) analyzed all the three different softwares and revealed despite the differences among the algorithms between different softwares, the outcome of most stable and least stable reference genes was largely comparable for each sample set.

During analysis of different datasets, we observed that *UBC*, *HSP90* and *GAPDH* exhibited most stable gene expression across heat and salt stress conditions and can be used as a common stable internal control for expression studies under the given abiotic stresses. In contrast, several studies identified stress specific stable housekeeping genes, used as an internal control (Barsalobres-Cavallari et al., 2009; Garg et al., 2010; Sinha et al., 2015). However, not only for experimental conditions, in some cases different algorithms identified different stable housekeeping genes during analysis of the same datasets with different programs (Van Hiel et al., 2009; Reddy et al., 2013; Lopez-Pardo et al., 2013).

Considering the results examined by different programs for heat stressed tissue samples (root, stem, and leaves), *UBC* (Ubiquitin C), *HSP90* (Heat Shock Protein 90), and *GAPDH* (glyceraldehyde-3-phosphate dehydrogenase) genes are the most stable genes across the tissues used in the study using geNorm and NormFinder. *UBC* and *HSP90* were identified as the two most stable genes for heat stress conditions using geNorm with *M-*value of 0.666 were similar to earlier identified housekeeping genes for chickpea datasets with *M-*value of 0.28 (Jain et al., 2006).

Identified housekeeping gene *UBC*, (*Ubiquitin C*) has been associated with DNA repair, cell cycle regulation, kinase modification, endocytosis, and regulation of other cell signaling pathways. The ubiquitin–proteasome system is a major non-lysosomal proteolytic pathway that functions constitutively to degrade abnormal or damaged proteins (Hegde et al., 1997). After analyzing the datasets for salinity stress conditions, the gene *GAPDH*, an enzyme of glycolysis (Giulietti et al., 2001) outperformed in comparison to all other genes, and can be used as internal control for qRT-PCR analysis. *GAPDH* was also found stable housekeeping gene during expression analysis across tissues and genotypes in sugarcane (Iskandar et al., 2004). *GAPDH* been identified as a central metabolism enzyme is an important energy-yielding step in carbohydrate metabolism and its ability to perform mechanistically different functions (Zimmer and Wen, 2013). Another identified gene, *HSP90* is known to play an important role in protein refolding in cells exposed to environmental stress and is required for the conformational maturation of several important signaling proteins (Jakob and Buchner, 1994). Additionally, *HSP90* has been shown role in the proteasome-dependent degradation of a selected group of cellular proteins (Whitesell et al., 1994). *Actin* is reported as one of the most commonly used housekeeping gene which is found to be essential for a range of cellular functions. Some of the major roles include cell division, migration, junction formation, chromatin remodeling, transcriptional regulation, vesicle trafficking, and cell shape regulation (Perrin and Ervasti, 2010).

Validation of identified most stable and the combination of stable genes in comparison to the most unstable and widely used genes revealed significant differences in the fold change expression when normalized with the targeted candidate genes. Gene expression showed enhanced expression level with *18Sr RNA* in the case of heat stress and *TUB6* for salt stress conditions. These results indicated their low stability in the gene expression under targeted stress conditions. The validation result revealed that normalization with the most stable housekeeping genes based on the ranking had a similar level of gene expression for targeted genes, *uspA*, and *uspB*. Therefore, for better accuracy during gene normalization studies, a combination of identified stable housekeeping genes should be used. Our findings signify the importance of identification of specific housekeeping genes for specific stress conditions. In the case of pigeonpea, now together with this study, we got reference genes as *IF4α* and *TUB6* for drought stress (Sinha et al., 2015), *UBC*, *HSP90*, and *GAPDH* for heat stress and *GAPDH*, *UBC*, and *HSP90* for salt stress.

## Conclusion

Our study identified *UBC*, *HSP90, GAPDH*, and *GAPDH, UBC*, *HSP90* as the most stable housekeeping genes under heat and salt stress conditions, respectively, for gene expression studies. Our data suggests that the expression of *18Sr RNA* is not very stable for heat stress condition, and for salt stress condition *TUB6* is the least stable gene. Validation of the identified stable housekeeping genes suggested that although, single reference gene gave reliable results, a combination of stable genes produces even better results for heat as well as salt stress conditions.

## Conflict of Interest Statement

The authors declare that the research was conducted in the absence of any commercial or financial relationships that could be construed as a potential conflict of interest.

## Footnotes

1 http://www.icrisat.org/gt-bt/iipg/Home.html

2 http://www.bioinformatics.nl/cgi-bin/primer3plus/primer3plus.cgi/

3 http://download.gene-quantification.info/

4 http://medgen.ugent.be/~jvdesomp/genorm/

5 http://moma.dk/normfinder-software

## Supplementary Material

FIGURE S1 | Gene expression analysis of candidate housekeeping genes across the tissues of heat and salt samples. This figure shows Ct distribution of each candidate reference genes among different samples of **(a)** heat and **(b)** salt tissues.

FIGURE S2 | Heat map of candidate genes for heat and salt stress samples. This figure **(a)** heat and **(b)** salt, shows a heat map of candidate genes plotted based on normalized Ct mean values. Clustering of genes was based upon the Ct mean values of individual candidate genes across tissues. The detailed description of samples is provide in supplementary table S1.

TABLE S1 | List of different tissue samples used for qRT-PCR analysis.

TABLE S2 | Descriptive statistics of candidate genes under heat and salt stress conditions using BestKeeper software.
