# Supplementary figures and images for: Selection and Validation of Housekeeping Genes as Reference for Gene Expression Studies in Pigeonpea (Cajanus cajan) under Heat and Salt Stress Conditions
Source: Front Plant Sci. 2015 Dec 21;6:1071. doi: 10.3389/fpls.2015.01071 (PMC4865767; doi:10.3389/fpls.2015.01071)

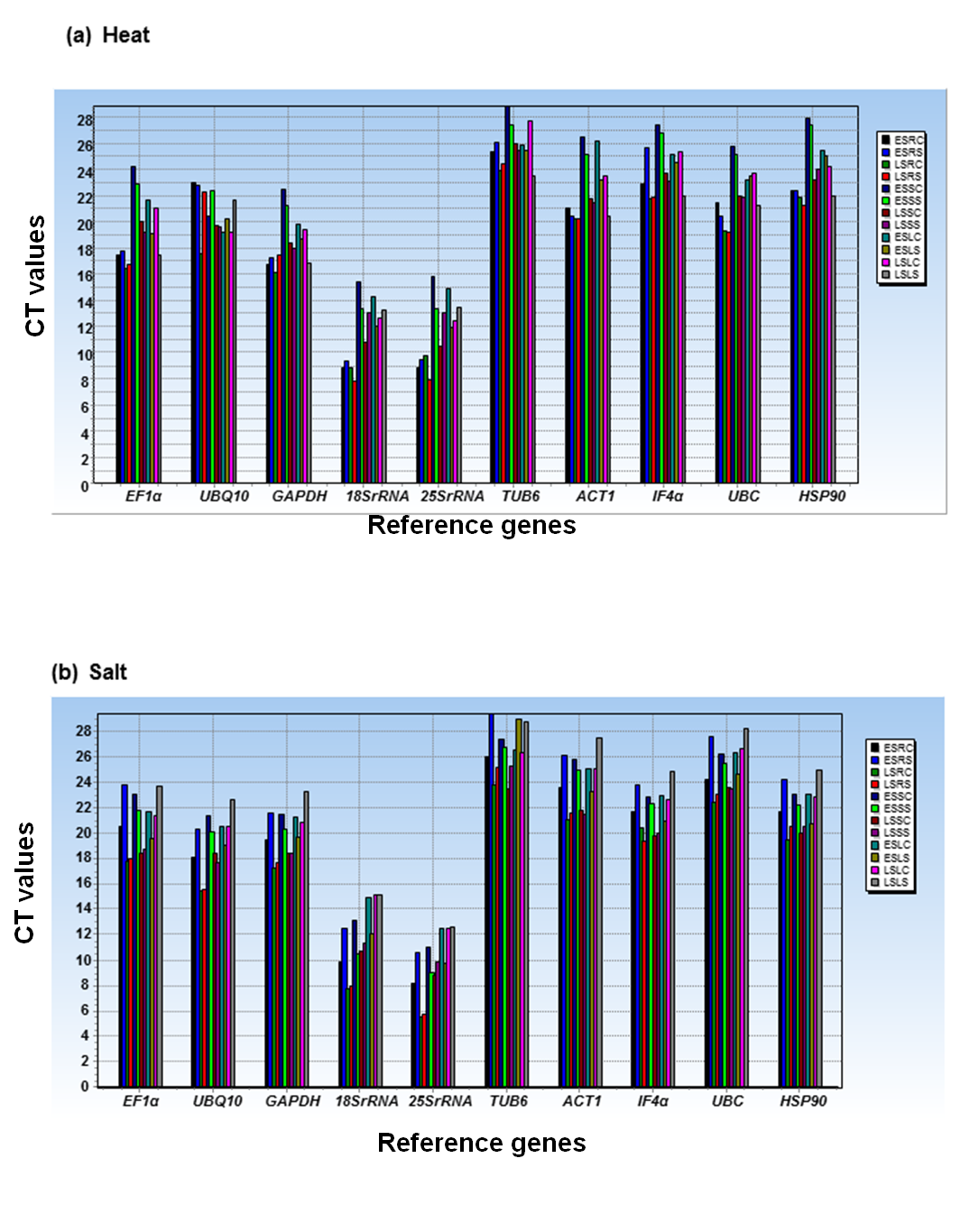

Supplement: Supplementary file 1 [file Image_1.TIF]

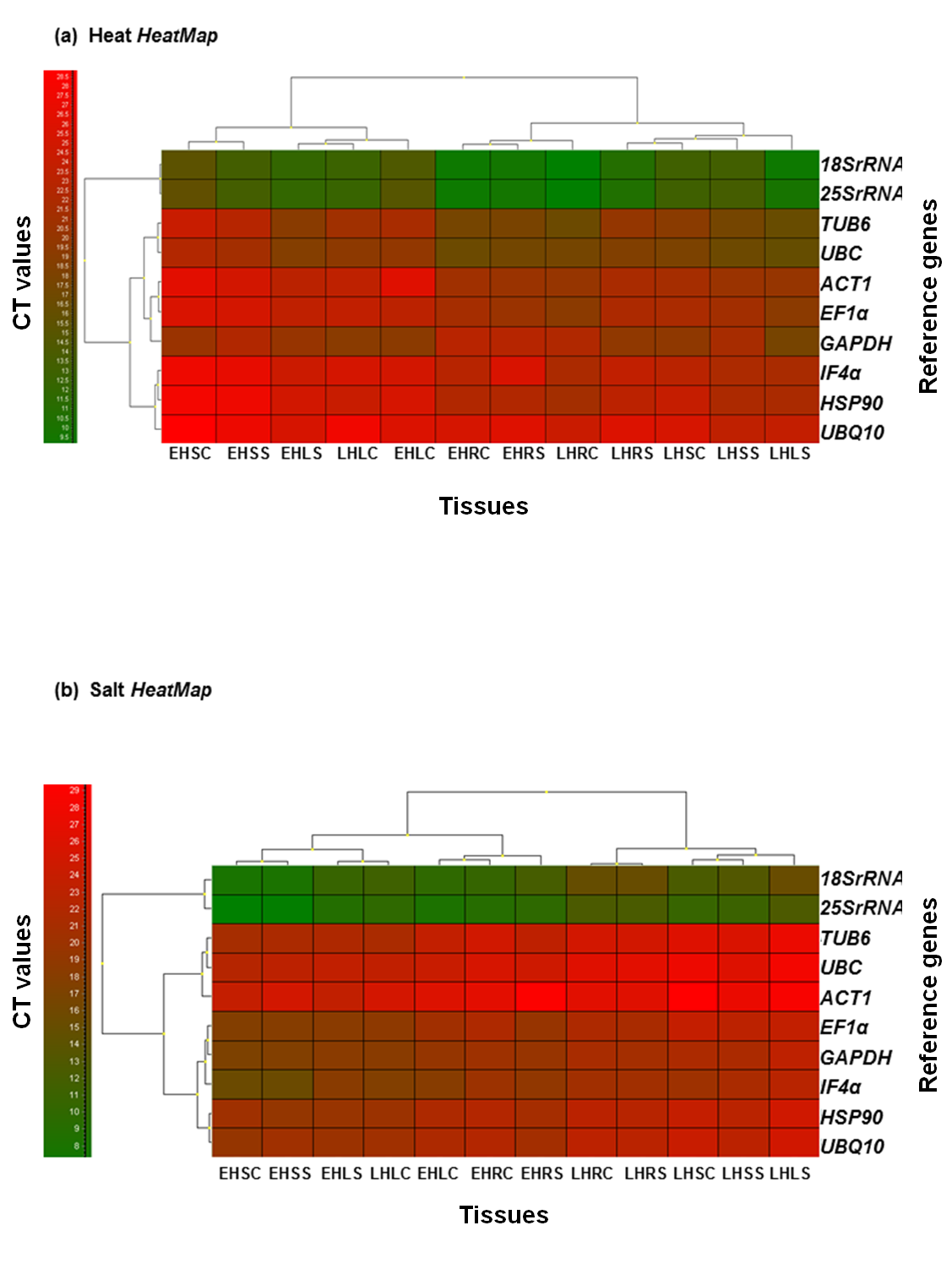

Supplement: Supplementary file 2 [file Image_2.TIF]
