# Supplementary material for: Selection and Validation of Housekeeping Genes as Reference for Gene Expression Studies in Pigeonpea (Cajanus cajan) under Heat and Salt Stress Conditions
Source: Front Plant Sci. 2015 Dec 21;6:1071. doi: 10.3389/fpls.2015.01071 (PMC4865767; doi:10.3389/fpls.2015.01071)
Supplement: Supplementary file 3 [file Table_1.DOCX]

**Table S1.** List of different tissue samples used for qRT-PCR analysis

| Sample no. | Sample code | Description^*^ |
| --- | --- | --- |
| *Heat samples* |  |  |
| 1 | EHRC | Early heat root control |
| 2 | EHRS | Early heat root stress |
| 3 | LHRC | Late heat root control |
| 4 | LHRS | Late heat root stress |
| 5 | EHSC | Early heat shoot control |
| 6 | EHSS | Early heat shoot stress |
| 7 | LHSC | Late heat shoot control |
| 8 | LHSS | Late heat shoot stress |
| 9 | EHLC | Early heat leaf control |
| 10 | EHLS | Early heat leaf stress |
| 11 | LHLC | Late heat leaf control |
| 12 | LHLS | Late heat leaf stress |
| *Salinity samples* |  |  |
| 13 | ESRC | Early salt root control |
| 14 | ESRS | Early salt root stress |
| 15 | LSRC | Late salt root control |
| 16 | LSRS | Late salt root stress |
| 17 | ESSC | Early salt shoot control |
| 18 | ESSS | Early salt shoot stress |
| 19 | LSSC | Late salt shoot control |
| 20 | LSSS | Late salt shoot stress |
| 21 | ESLC | Early salt leaf control |
| 22 | ESLS | Early salt leaf stress |
| 23 | LSLC | Late salt leaf control |
| 24 | LSLS | Late salt leaf stress |

*Early and late denotes for vegetative and reproductive stage respectively.
