# Supplementary material for: Selection and Validation of Housekeeping Genes as Reference for Gene Expression Studies in Pigeonpea (Cajanus cajan) under Heat and Salt Stress Conditions
Source: Front Plant Sci. 2015 Dec 21;6:1071. doi: 10.3389/fpls.2015.01071 (PMC4865767; doi:10.3389/fpls.2015.01071)
Supplement: Supplementary file 4 [file Table_2.DOCX]

**Table S2**. Descriptive statistics of candidate genes under heat and salt stress conditions using BestKeeper software

| Factor | Candidate genes | | | | | | | | | |
| --- | --- | --- | --- | --- | --- | --- | --- | --- | --- | --- |
|  | *EF1α* | *UBQ10* | *GAPDH* | *18SrRNA* | *25SrRNA* | *TUB6* | *ACT1* | *IF4α* | *UBC* | *HSP90* |
| *Heat samples* | | | | | | | | | | |
| GM | 19.34 | 20.60 | 18.46 | 11.40 | 11.54 | 25.77 | 22.38 | 24.09 | 22.13 | 23.84 |
| AM | 19.49 | 20.67 | 18.54 | 11.65 | 11.79 | 25.81 | 22.49 | 24.16 | 22.22 | 23.93 |
| Min | 16.41 | 17.58 | 16.12 | 7.82 | 7.91 | 23.54 | 20.20 | 21.72 | 19.23 | 21.25 |
| Max | 24.26 | 22.97 | 22.46 | 15.37 | 15.84 | 28.82 | 26.46 | 27.41 | 25.70 | 27.90 |
| SD | 2.04 | 1.45 | 1.49 | 2.08 | 2.08 | 1.13 | 1.99 | 1.64 | 1.69 | 1.74 |
| CV | 10.48 | 7.01 | 8.04 | 17.83 | 17.68 | 4.39 | 8.85 | 6.78 | 7.61 | 7.29 |
| Min [x-fold] | -7.64 | -8.10 | -5.05 | -11.98 | -12.37 | -4.69 | -4.56 | -5.18 | -7.50 | -6.02 |
| Max [x-fold] | 30.15 | 5.17 | 16.07 | 15.64 | 19.66 | 8.27 | 16.92 | 9.96 | 11.83 | 16.68 |
| SD [± x-fold] | 4.12 | 2.73 | 2.81 | 4.22 | 4.24 | 2.19 | 3.97 | 3.11 | 3.23 | 3.35 |
|  |  |  |  |  |  |  |  |  |  |  |
| *Salt samples* | | | | | | | | | | |
| GM | 20.60 | 19.03 | 19.87 | 11.47 | 9.36 | 26.43 | 23.85 | 21.72 | 25.10 | 21.88 |
| AM | 20.70 | 19.15 | 19.95 | 11.74 | 9.67 | 26.49 | 23.93 | 21.79 | 25.17 | 21.94 |
| Min | 17.79 | 15.48 | 17.22 | 7.80 | 5.48 | 23.51 | 21.04 | 19.31 | 22.39 | 19.46 |
| Max | 23.80 | 22.68 | 23.25 | 15.18 | 12.65 | 29.35 | 27.53 | 24.83 | 28.25 | 24.97 |
| SD | 1.85 | 1.78 | 1.49 | 2.06 | 1.86 | 1.47 | 1.83 | 1.44 | 1.59 | 1.46 |
| CV | 8.92 | 9.31 | 7.46 | 17.58 | 19.27 | 5.56 | 7.63 | 6.62 | 6.30 | 6.64 |
| Min [x-fold] | -7.00 | -11.71 | -6.28 | -12.78 | -14.66 | -7.55 | -7.02 | -5.32 | -6.57 | -5.33 |
| Max [x-fold] | 9.20 | 12.57 | 10.41 | 13.08 | 9.79 | 7.60 | 12.81 | 8.62 | 8.82 | 8.55 |
| SD [± x-fold] | 3.60 | 3.44 | 2.80 | 4.18 | 3.64 | 2.78 | 3.55 | 2.72 | 3.00 | 2.75 |

Abbreviations: GM: geometric mean; AM: athematic mean; Min and Max: threshold values; SD: standard deviation; CV: coefficient of variation; Max [x-fold] and Min [x-fold]: threshold expression levels expressed as absolute x-fold over or under regulated coefficient; SD [± x-fold] standard deviation of absolute regulation coefficient
